# The Clinical, Diagnostic, Therapeutic, and Prognostic Characteristics of Brain Metastases in Prostate Cancer: A Systematic Review

**DOI:** 10.1155/2022/5324600

**Published:** 2022-11-27

**Authors:** Seyyedmohammadsadeq Mirmoeeni, Amirhossein Azari Jafari, Muffaqam Shah, Fateme Salemi, Seyedeh Zohreh Hashemi, Ali Seifi

**Affiliations:** ^1^School of Medicine, Shahroud University of Medical Sciences, Shahroud, Iran; ^2^Deccan College of Medical Sciences, P.O. Kanchanbagh, DMRL ‘X' Road, Santhosh Nagar, Hyderabad 500058, Telangana, India; ^3^School of Medicine, Islamic Azad University of Medical Sciences, Yazd, Iran; ^4^Researcher at the Research Center of Tehran University of Medical Sciences, Pharmacology Department, Tehran, Iran; ^5^Department of Neurosurgery, Division of Neuro Critical Care, University of Texas Health Science Center at San Antonio School of Medicine, San Antonio, TX, USA

## Abstract

**Aim:**

Prostate cancer (PCa) is the second most common nonskin malignancy and the second most common cause of cancer-related deaths in men. The most common site of metastasis in PCa is the axial skeleton which may lead to back pain or pathological fractures. Hematogenous spread to the brain and involvement of the central nervous system (CNS) are a rare occurrence. However, failed androgen deprivation therapy (ADT) may facilitate such a spread resulting in an advanced metastatic stage of PCa, which carries a poor prognosis.

**Methods:**

In this systematic review, we searched the PubMed, Scopus, and Web of Science online databases based on the PRISMA guideline and used all the medical subject headings (MeSH) in terms of the following search line: (“Brain Neoplasms” OR “Central Nervous System Neoplasms”) and (“Prostatic Neoplasms” OR “Prostate”). Related studies were identified and reviewed.

**Results:**

A total of 59 eligible studies (902 patients) were included in this systematic review. In order to gain a deeper understanding, we extracted and presented the data from included articles based on clinical manifestations, diagnostic methods, therapeutic approaches, and prognostic status of PCa patients having BMs.

**Conclusion:**

We have demonstrated the current knowledge regarding the mechanism, clinical manifestations, diagnostic methods, therapeutic approaches, and prognosis of BMs in PCa. These data shed more light on the way to help clinicians and physicians to understand, diagnose, and manage BMs in PCa patients better.

## 1. Introduction

Prostate cancer (PCa) is the second most common nonskin malignancy and the second most common cause of cancer-related deaths in men [1–3]. PCa is a clinically heterogeneous cancer that develops and progresses through various stages. These can range from initial prostatic intraepithelial neoplasia to metastatic disease, as well as hormone-refractory disease [1, 3]. It is demonstrated that several environmental and genetic risk factors such as age, genetic mutations, race/ethnicity, family history, lifestyle, and diet can strongly impact the progression of PCa [1, 4, 5]. PCa can often be asymptomatic, however, the most common signs and symptoms are difficulty in micturition, straining to start, increased frequency, and nocturia [2]. Surgery and radiotherapy are the established standard treatments of PCa. However, patients in whom such treatments prove unsuccessful are mainly treated with androgen deprivation therapy (ADT), which works by shrinking androgen-dependent tumors. A possible consequence of failed ADT is the subsequent development of recurrent androgen-independent PCa, with brain metastases (BMs) and reduced cognitive functions [3, 6, 7].

The most common site of metastasis in PCa is the axial skeleton which may lead to back pain or pathological fractures [2]. Hematogenous spread to the brain and involvement of the central nervous system (CNS) are a rare occurrence. However, failed ADT may facilitate such a spread resulting in an advanced metastatic stage of PCa, which carries a poor prognosis. This occurs mostly in intracranial sites such as the leptomeninges, cerebrum, and cerebellum, with many of the nonfocal neurologic symptoms being attributed to intracranial hypertension [6, 8–13]. It has been demonstrated that patients with nonadenocarcinoma PCa have a higher chance of BMs [10]. Recently, there has been a trend showing an increase in the number of PCa cases with a reported metastatic brain lesion. This brings to light the numerous challenges we face in terms of properly understanding, diagnosing, and managing PCa patients with BMs [14–33]. Given the lack of knowledge regarding the issue of metastatic brain lesions in PCa, we decided to comprehensively and systematically review the latest evidence regarding the clinical manifestations, diagnosis, treatment, and prognosis of the BMs in PCa.

## 2. Methods

### 2.1. Search Strategy

This systematic review was conducted based on the Preferred Reporting Items for Systematic Reviews and Meta-Analyses (PRISMA) guidelines [34]. Two researchers (S. M and A. AJ) independently searched the PubMed, Scopus, and Web of Science online databases using all the medical subject headings (MeSH) in terms of the following search line: (“Brain Neoplasms” OR “Central Nervous System Neoplasms”) and (“Prostatic Neoplasms” OR “Prostate”) until November 7^th^, 2021, in the title and abstract and without any date or language restrictions. The intention of this systematic search was to search and find studies reporting clinical manifestations, diagnostic approaches, treatments, and prognosis of BMs in PCa patients. Moreover, to find any studies which are not resulted through the online searches, we performed a manual hand-searching process to identify and include any further relevant studies.

### 2.2. Study Selection

**Figure 1:**
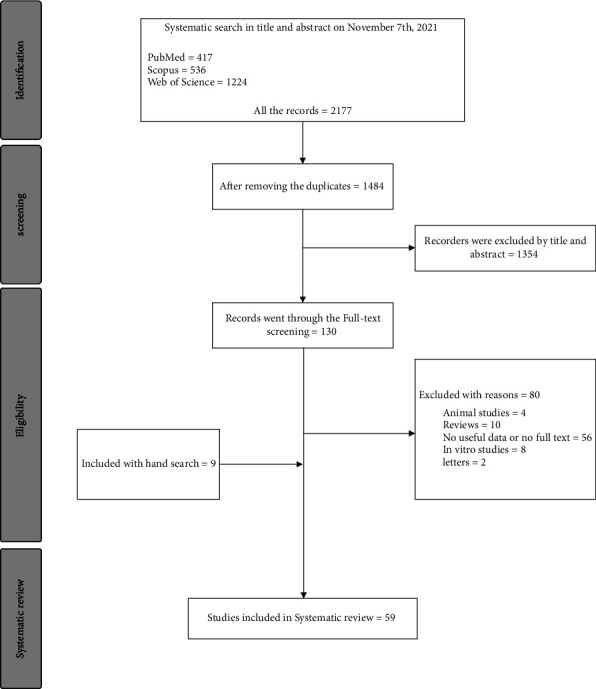
The PRISMA flow diagram of study selection.

As described in Figure 1, all the records resulting from the systematic search underwent a screening assessment separately by two independent researchers (S. M and A. AJ). Subsequent to removing the duplicate records, the researchers assessed the articles through screening by title and abstract, and sequentially in the final stage, they reassessed the studies using full-text screening. At this stage, we excluded studies meeting our exclusion criteria such as clinical trials, letters, reviews, animal studies, *in vitro* studies, articles without any useful data, studies reporting other metastases from PCa, and studies reporting BMs originated from other cancers. In case of any discrepancies and conflicts about studies, they were resolved by discussion with the third researcher (A. S). In the end, 59 eligible articles met the inclusion criteria, and therefore, they were included in this systematic review. The PRISMA process of this study is presented in detail in Figure 1.

### 2.3. Exclusion and Inclusion Criteria

Our main inclusion criteria included observational studies such as cohort studies, cross-sectional studies, and case series, and case report studies that reported diagnostic approaches, clinical manifestations, available treatments, and prognosis of BMs in PCa patients.

Our exclusion criteria included (1) articles reporting BMs originated from other cancers, (2) articles reporting other organ metastases from PCa instead of the brain, and (3) clinical trials, letters, reviews, animal studies, *in vitro* studies, and articles without any useful data or without any available full text.

### 2.4. Quality Assessment

As shown in Table 1, we assessed the methodological quality of all included studies independently by two researchers (S. M and A. AJ) using the NIH quality assessment tool for observational studies [35].

### 2.5. Data Extraction

Two independent researchers (S. M and A. AJ) extracted all the intended data from the final eligible articles, and in terms of any disagreement, they consulted with the third researcher (A. S). For each included study, the authors' names, publication year, type of study, total sample size, type of PCa, all the reported clinical manifestations, diagnostic methods, treatments, and prognosis status.

## 3. Results

### 3.1. Demographic Information

Following the completion of the study selection process, as shown in Figure 1, a total of 59 eligible studies [9, 10, 13–24, 26–30, 32, 36–74] (902 PCa patients with brain BMs) were included in this systematic review. Details regarding the included studies such as the year of publication, type of the study (case report or case series), sample size, and the quality assessment are presented in Table 1.

In order to gain a deeper understanding, we extracted and presented the data from included articles based on the clinical manifestations, diagnostic methods, therapeutic approaches, and prognostic status of PCa patients having BMs.

### 3.2. Clinical Manifestations and Diagnosis Methods

Fifty-seven [9, 10, 13–24, 26–30, 32, 36–39, 41, 43–74] out of the 59 studies including 852 patients had reported BMs in PCa patients presenting with several clinical manifestations ranging from general symptoms such as hematuria, increased urinary frequency, and weakness to neurologic signs and symptoms such as aphasia, dysphasia, dysarthria, hemiplegia, headache, dizziness, confusion, double vision, visual field cut, ataxia, seizures, delirium, dementia, loss of appetite, and even behavioral changes. Moreover, different diagnostic methods including prostate-specific antigen (PSA), brain computed tomography (CT), brain magnetic resonance imaging (MRI), bone scan, multispectral immunofluorescence, immunohistochemistry (IHC), DNA sequencing, positron emission tomography (PET), prostate biopsy, and CSF analysis were used to diagnose BM in patients with PCa. More information regarding the clinical manifestations and diagnostic methods used for these patients is presented in Table 2.

### 3.3. Therapeutic Approaches and Prognosis Status

As shown in Table 3, 901 patients from fifty-eight [9, 10, 13–24, 26–30, 32, 36–40, 42–74] out of the 59 studies had demonstrated varied therapeutic approaches such as immunotherapy, ADT, whole-brain radiation therapy (WBRT), craniotomy, prostatectomy, adjuvant radiation therapy, docetaxel chemotherapy, and medications such as abiraterone, prednisone, pembrolizumab, dexamethasone, bicalutamide, and leuprorelin. Additionally, the prognostic overview of the PCa patients developing BMs was not promising. Most of the cases passed away despite the fact that they received different kinds of treatments ranging from radiotherapy, chemotherapy, surgery, and even immunotherapy.

## 4. Discussion

Metastatic lesions in the brain arising from lung cancer (∼30% of patients), breast cancer (∼30%), and melanoma (∼45%) are relatively common and well-studied. There is, however, scarceness of information regarding BMs arising from PCa, due to its rarity. Reports in the literature estimate the incidence to be between 0.16% and 0.63%, with a median survival time after BMs detection between 2.8 and 4.5 months [13].

In a manner similar to other neoplasia's, there is a progression from initial normal prostatic epithelium to intraepithelial neoplasia, which can lead to either localized adenocarcinoma, squamous carcinoma, neuroendocrine carcinoma, or a combination of the above. Continued neoplastic change leads to eventual metastatic spread, once the basal layer/basement membrane has been breached [75]. It has been mentioned that prostate small cell carcinoma seems to have a greater tendency to produce BMs compared to prostate adenocarcinoma [64].]

**Figure 2:**
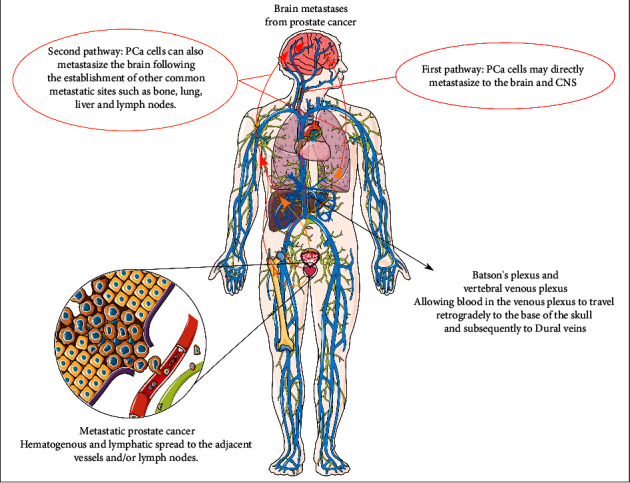
The pathways and mechanisms of brain metastases in prostate cancer (this figure was drawn using images from Servier medical art (https://smart.servier.com) licensed by a Creative Commons Attribution 3.0 unported license.)

Various theoretical mechanisms have been proposed for the CNS metastases of PCa. As shown in Figure 2, these can be broadly classified as the hematogenous and lymphatic spread and severe impairment of the immune system leading to a breakdown of the blood barrier and the soil-and-seed and epithelial-to-mesenchymal transition theory, as well as the multistep or cascade process theory [76–78]. After a wide and thorough review of the available evidence, it is evident that the true nature of PCa varies from case to case. Thus, it is a complex interaction among the numerous multiple mechanisms listed above.

The broad concept consists of tumor cells detaching from the primary tumor mass to invade the basement membrane and the surrounding microenvironment. They intravasate into either the surrounding blood vessels or lymphatics. They then synthesize proangiogenic factors that will initiate neoangiogenesis. This is followed by extravasation at the secondary sites, the formation of micrometastases, and the subsequent process of metastatic colonization [79].

### 4.1. Clinical Manifestations

The neurological signs and symptoms of BMs in PCa lesions are usually related to the consequent intracranial hypertension [76]. Additionally, issues also arise based on the specific area of localization.

As described in Table 2, these include symptoms such as headache, nausea, vomiting, seizures, confusion, weakness, aphasia, visual disturbances, ataxia, motor dysfunction, mono/hemiparesis, mental status or behavioral changes, cranial nerve dysfunction, and delirium. It was also demonstrated that patients can be asymptomatic, and the diagnosis of BMs in PCa may be an incidental finding during other investigations [9, 10, 13–24, 26–30, 32, 36–39, 41, 43–74, 76, 77].

Prostate neoplastic cells rarely involve the brain stem and cerebellum, therefore, focal neurologic presentations including seizures and ataxia are uncommon. As we showed in the results, most patients show unspecific neurologic symptoms including headaches and papilledema, which can be explained as being caused by elevated intracranial pressure and frontal lobe syndrome. However, in some rare cases, PCa BMs are asymptomatic. PCa patients with BMs are often in the end stages of the disease, or they may also have other accompanying chronic illnesses such as atherosclerosis, which may hide their CNS involvement and make the diagnosis even more challenging. Due to these multiple factors, cranial nerve and pituitary tumor spread, tumor mass effect, and meningeal involvement with hemorrhage of the contiguous brain tissue are frequently missed in these patients [9, 10, 13–24, 26–30, 32, 36–39, 41, 43–74, 76, 77, 80–83].

### 4.2. Diagnostic Approaches to the BMs of PCa

Considering the findings, the brain involvement acts as an uncommon yet serious presentation of PCa notably in patients with widespread metastases and multiorgan tumor spread. It has significant implications on patient prognosis and overall survival. Hence, the necessity is for early diagnosis and management, especially in patients with high clinical suspicion for metastases [36–40, 82, 84]. As shown in Table 2, several diagnostic approaches were used in BMs of PCa [9, 10, 13–24, 26–30, 32, 36–39, 41, 43–74]. Herein, we categorized and discussed them separately based on the nature of the diagnostic approach.

#### 4.2.1. Imaging Modalities

Due to the impacts of CNS involvement on PCa, staging imaging is indicated in specific clinical situations. MRI and CT are the modalities which are chosen to detect brain involvement in systemic malignancies with or without neurologic symptoms. Moreover, imaging is also indicated in patients with neurological symptoms without signs of malignancy [37, 39, 44, 85–88].

In metastatic PCa, routine radiologic screening is performed to detect asymptomatic brain invasion, since tumors spreading to the lymph nodes and bone are more likely to have intracranial metastases [89]. In regards to cost-effectiveness, Hatzoglou et al. mentioned that screening asymptomatic PCa patients with BMs for early detection may warrant further studies before it can become a routine practice [10]. Due to the low incidence of BMs in PCa, MRI is not indicated in asymptomatic patients or those with mild neurological symptoms [76]. However, increased incidence of metastatic PCa, especially in those with neurological presentations, requires screening guidelines to reduce mortality, morbidity, and treatment costs, as well as to improve overall patient survival [9, 10, 90].

As illustrated in the results, the first line imaging modality to detect BMs in PCa is a noncontrast CT scan which identifies lethal neurological conditions including hemorrhage, hydrocephaly, or massive compressive lesions [9, 29, 30, 38, 41, 44, 87]. Before using MRI as a diagnostic tool for detecting CNS metastases, imaging modalities lack the sensitivity to identify multiple intraparenchymal metastatic lesions. Therefore, most BMs were reported as unifocal [9, 73, 91].

Some PCa patients present brain hemorrhages as a result of tumor invasion which appears as hyperdense lesions on CT scans. However, nonhemorrhagic spots demonstrate variable densities compared with the surrounding brain tissue [10]. Calcification can make the diagnosis of BMs in PCa unlikely, as well as other malignancies [86, 92].

Before the development of advanced MRI techniques, contrast-enhanced CT scan was the second diagnostic option to confirm BMs in challenging PCa cases. PCa metastases may appear as solid, cystic, nodular, mixed, or ring-like enhancements in this modality [93–95].

Nowadays, MRI is the gold standard diagnostic option to identify malignant CNS lesions in PCa, and it can also be used to follow-up the patients during the course of treatment. Advanced techniques/sequences such as magnetic resonance spectroscopy (MRS), magnetic resonance perfusion (MRP), diffusion-weighted imaging (DWI), and diffusion tensor imaging (DTI) are advantageous due to them having a higher sensitivity in order to better differentiate BMs in PCa from other CNS malignant or nonmalignant lesions [86]. MRI is indicated to find any possible tumoral lesion which may have been missed/undiagnosed on the CT scan. It is used before surgery, radiosurgery, or when an underlying malignancy is suspected. In spite of a CT scan, MRI can differentiate a malignant metastatic lesion from a noncancerous pathology such as an infection [88]. In *T*1, signal nonhemorrhagic BMs appears isointense or hypointense, and metastatic hemorrhagic lesions are hyperintense. In *T*2 weighted MRI, tumoral masses show hyperintense signals [86, 96, 97]. Nevertheless, BMs in PCa show unspecific and variable signs in MRI, making it difficult to differentiate it from other brain lesions, including metastases from other cancers, and primary brain tumors such as gliomas, or even infectious masses [44].

Most metastatic brain tumors including those from PCa are unifocal, however, multifocal lesions are reported in some cases. Malignant cells often invade the brain tissue at the junction between gray and white matter, particularly at the areas which border the main arteries supplying the brain with blood [98, 99]. Besides involving the cerebellum, PCa cells tend to involve the posterior fossa [100, 101]. It is suggested that the adjacent structures of brain parenchyma such as the skull and meninges might also become involved [102]. 18 fluorodeoxyglucose positron emission tomography (FDG-PET) and other advanced molecular imaging techniques may also help in diagnosing more challenging cases [86].

#### 4.2.2. Histopathology

Based on our findings showing that biopsy and tissue assessment was used in most cases, histopathology is the most accurate test for diagnosing CNS metastases in PCa, although, its invasive nature and side effects outweigh the benefits, especially for end-stage or critically ill PCa patients. Therefore, the tissue assessment of other distant metastases of PCa or brain imaging may act as substitutes to diagnose neurologic involvement in most patients [9, 10, 14, 18–20, 36, 43–46, 49, 57, 60–62, 64, 66, 69, 70, 72, 73].

Most brain metastatic lesions originate from prostate adenocarcinoma since it is the most common type of PCa. However, other less frequent histological subtypes including small cell carcinoma and neuroendocrine carcinoma of the prostate have the highest tendency to cause brain involvement in a shorter period. Besides tumor staging, histology plays a notable role in the spread of PCa to the CNS [10, 103]. Hematoxylin and eosin staining of BMs in prostate adenocarcinoma demonstrate glandular patterns that are PSA-positive in immunohistochemistry [10].

Memorial Sloan Kettering Cancer Center evaluated the gross pathology of BMs in deceased PCa patients and found hemorrhagic metastases in some autopsies. Like primary CNS neoplasms, the metastatic masses of malignant prostate cells exhibited distinct borders surrounded by vasogenic edema [10].

#### 4.2.3. Molecular and Genetic Biomarkers

Of particular interest is one of our results which demonstrated that progressively higher reelin levels have been found in 39% of PCa patients. Reelin is an extracellular glycoprotein that is involved in regulating neuronal migration during brain development and also in cancer biology. Thus, it can be suggested that reelin levels could be used as a marker to predict the aggressiveness of prostatic cells [77]. Another finding of interest showed that the brain involvement of PCa should be assessed in high-risk patients; also understanding molecular characteristics of malignant cells might help us in the diagnosis and management process [9]. In this regard, a study found that forty percent of patients with prostate tumors demonstrated mild ADAM metallopeptidase with thrombospondin type 1 motif 13 (ADAMTS-13) deficiency. Low ADAMTS-13 activity could result in elevated levels of highly polymeric von Willebrand factor (vWF), which might facilitate adhesive interactions with both circulating tumor cells and platelets, resulting in thrombus formation and the presumptive development of a metastatic colony [104].

### 4.3. Treatments of BMs in PCa

Due to more efficient treatment facilities and earlier diagnosis, the prognosis of patients with PCa has become more favorable. Therefore, the rate of rare complications such as BMs in PCa has become more common [90, 105, 106]. Hence, it becomes necessary to study and find more effective therapeutic approaches for cases of BMs in patients with PCa. As shown in Table 3, many diverse classes of treatments were administrated in BMs of PCa. Selected therapies for BMs can be divided into two general categories, main therapies and supportive therapies [9, 10, 13–24, 26–30, 32, 36–40, 42–74, 107].

We demonstrated that supportive therapies may reduce and alleviate the symptoms of cerebral metastases which typically include anticoagulants, antiepileptic drugs (AEDs), and corticosteroids. The main treatments that are considered directly for tumor management usually include chemotherapy, radiotherapy, and surgery [9, 107]. Corticosteroids, especially dexamethasone, are used to treat cerebral edema and any symptoms in patients with BMs, reporting a 75% reduction in neurological symptoms. Dexamethasone is usually given in two separate doses of 4 to 8 mg daily, according to the instructions, and higher doses are used only in more severe cases or if there is no effective response within 48 hours after administration [60, 73, 88, 108, 109]. However, corticosteroids have no place in asymptomatic patients. In some studies, it is recommended that dexamethasone can be prescribed to reduce the incidence of acute radiation toxicity if patients were asymptomatic and showed signs of cerebral edema on radiographic imaging before the radiotherapy [110]. In one study, mannitol, furosemide, and dexamethasone as supportive therapy were used to reduce intracerebral pressure, and have since started as major therapies for the treatment of BMs such as WBRT [29].

The most effective main treatments in the management of BMs are considered based on the number and type of BMs, the type of primary tumor, the location of the BMs, the rate of spread and control of the disease, and how the patient responds to treatment [107].

Our findings suggest that radiotherapy is currently used as a common treatment in most patients with BMs [9, 14, 17, 18, 21, 27, 29, 30, 36, 37, 43, 44, 47, 51, 55, 58, 60, 61, 63, 67, 71, 111]. In this regard, a study by Sita et al. focusing on radiotherapy in BMs following PCa provides a significant breakthrough for radiotherapy-prioritized treatment patterns. It suggests that five treatment approaches could be offered for comorbid radiotherapy treatments, which include WBRT, stereotactic radiosurgery (SRS), the base of skull radiotherapy, concurrent cabazitaxel plus WBRT, and surgery followed by adjuvant WBRT. This study has demonstrated that the survival rate of patients undergoing WBRT increased from 4 to 9 months, and for SRS, it increased from 9 to 13 months [112].

Additionally, we showed the investigation of 31 PCa patients who experienced BMs and underwent the treatment with radiosurgery, and there was a reported increased life expectancy from 1.2 to 4.6 months. Moreover, patients who received surgical resection plus radiotherapy had an elevated survival rate from 1.2 to 13 months [13]. Also, during the follow-up of three PCa patients with brain involvement, surgical treatment was considered as the initial treatment, but the systemic status of the cancer was out of control and the number of metastases was more than 5, and SRS has not been considered because it cannot affect the whole disease, so all three patients were treated with WBRT only. The WBRT reduced the symptoms of BMs in all three patients, however, its effect on the prognosis of patients was not promising [29, 113]. Because elderly patients do not often tolerate standard radiotherapy treatments, personalized treatments can be a good option in managing elderly patients [29, 113].

Our findings showed that solitary BMs can be also treated with resection surgery followed by radiotherapy, specially WBRT [17]. In confirmation of that, another study demonstrated that a solitary BMs was reported in a 63-year-old patient with PCa, craniotomy with gross total resection of the tumor was performed and was followed by adjuvant WBRT. In this patient, during the 23-monthfollow-up after surgery, they saw an unrecognizable decrease in PSA levels with no evidence of recurrence [22]. Another patient was diagnosed with metastatic prostate adenocarcinoma who underwent craniotomy with biopsy and resection of the mass lesion simultaneously and then was subsequently treated with SRS due to the limited volume of involvement. After 7 months of follow-up, no recurrence of the lesion was reported [21]. Another elderly patient with a history of prostate adenocarcinoma highlighted the possibility of intracranial metastases of the prostate based on neurological symptoms. Due to his disability and poor prognosis, the patient preferred palliative treatment rather than radiotherapy. Although acute neurological symptoms improved after 3 days of dexamethasone use, the patient died 5 months after being diagnosed with BMs [26].

### 4.4. Prognosis and Mortality

We found that despite being devoted to PCa, BMs is rare and uncommon [9, 10, 13, 27, 112, 114–116], and the main survival rate estimations suggest that BMs in PCa has a poor prognosis [10, 112, 116, 117]. As presented in Table 3, many studies have been conducted to measure the survival rate and the mortality rate of PCa with BMs, for instance, it is reported in one study that the mean survival rate for only BMs in PCa is less than 28 months [9, 10, 13–24, 26–30, 32, 36–74, 117]. Sita et al. [112] has also demonstrated that the median survival for intracranial metastases in PC is 4 to 13 months. Moreover, it is established that the prognosis of parenchymal BMs is poor, with a mean survival rate of 1 to 7.6 months [116]. Tremont-Lukats IW and colleagues have reported that the overall median survival rate of PCa patients with BMs was 1 month from the time of diagnosis of CNS involvement; however, they suggested that undergoing the radiotherapy treatment can increase their median survival rate up to 3.5 months [9].

On the other hand, Cagney et al. [118] in a cohort study indicated that the median survival of BMs just in PCa patients was 12 months which was more than other primary cancers such as breast, small cell lung, and melanoma. One study found the Karnofsky performance score (KPS) to be effective in estimating the prognosis of elderly patients with BMs in PCa treated with WBRT. This study reported that patients with more than 70% of KPS are good candidates for treatment with long-term courses with WBRT [42].

Thus, in this review, we cannot compare the prognosis and the survival rate of BMs following PCa with other types of cancers. As diagnostic factors can play a major role in discovering the BMs in PCa, prognostic agents can also be considered crucial and even necessary in the prediction of disease prognosis. Malignant brain tumor domain-containing protein 1 (MBTD1) can play a critical role as a novel prognostic or diagnostic factor. As its overexpression is associated with poor prognosis and consequently, short survival time [119].

## 5. Conclusion

The intended purpose of conducting this systematic review was to gather and highlight all the current knowledge of BMs in PCa patients. We emphasized the importance of all the basic and clinical aspects of BMs in PCa. We have discussed the possible risk factors and mechanisms causing the BMs in PCa and then, we have described the available clinical approaches and diagnostic methods that can be used by physicians to identify the BMs in PCa patients and differentiate it from other neurological diseases. We have also demonstrated the therapeutic guidelines and treatments in BMs in PCa and along with the prognostic status of BMs in PCa. These data shed more light on the way to help clinicians and physicians to understand, diagnose, and manage BMs in PCa patients better.

## Figures and Tables

**Table 1:** The demographic information of all included studies reporting BMs in PCa.

Author	Year of publication	Type of studies	Total sample size	Quality assessment (good, fair, or poor)	Reference
Sena et al.	2021	Case report	1	Good	[36]
Pikis et al.	2021	Case series	46	Good	[37]
Parihar et al.	2021	Case report	1	Good	[38]
Hung et al.	2021	Retrospective study	204	Good	[39]
Boxley et al.	2021	Retrospective study	29	Fair	[40]
Son et al.	2020	Case report	1	Fair	[30]
Shida et al.	2020	Case report	3	Good	[29]
Saadatpour et al.	2020	Case report	1	Fair	[41]
Ross et al.	2020	Case report	1	Good	[28]
Nguyen et al.	2020	Retrospective cohort	21	Fair	[42]
Marchand Crety et al.	2020	Case report	1	Good	[27]
Liu et al.	2020	Case report	1	Good	[26]
Janda et al.	2020	Case report	1	Good	[24]
Bhambhvani et al.	2020	Retrospective cohort	31	Good	[13]
Aljarba et al.	2020	Case report	1	Good	[32]
Ahmad et al.	2020	Case report	1	Good	[43]
Kosaka et al.	2019	Case report	1	Fair	[23]
Kanyilmaz et al.	2019	Retrospective cohort	339	Good	[44]
Ishizaki et al.	2019	Case report	1	Good	[22]
Hogan et al.	2019	Case report	1	Good	[45]
Zanatta et al.	2018	Case report	1	Good	[46]
Reinas et al.	2018	Case report	1	Fair	[47]
Nunno et al.	2018	Case report	1	Fair	[48]
Jack et al.	2018	Case report	1	Good	[49]
Campagna et al.	2018	Case report	1	Fair	[21]
Watanabe et al.	2017	Case report	1	Good	[20]
Lam et al.	2017	Case report	2	Fair	[19]
Chang et al.	2017	Case report	1	Good	[18]
Guraya et al.	2016	Case report	1	Good	[50]
Barakat et al.	2016	Case report	1	Good	[17]
Mandaliya et al.	2015	Case report	2	Good	[51]
Hutton et al.	2015	Case report	1	Fair	[16]
Gajewska et al.	2015	Case report	1	Fair	[15]
Craig et al.	2015	Case report	1	Good	[14]
Hatzoglou et al.	2014	Retrospective cohort	21	Good	[10]
Placido et al.	2014	Case report	3	Fair	[52]
Hazell et al.	2013	Case report	4	Good	[53]
Caffo et al.	2013	Cohort	9	Fair	[54]
Caffo et al.	2012	Case series	22	Good	[55]
Flannery et al.	2010	Case series	10	Good	[56]
Yamada et al.	2009	Case report	1	Good	[57]
Sweets et al.	2009	Case report	1	Good	[58]
Kim et al.	2008	Case series	5	Good	[59]
Grenader et al.	2007	Case report	1	Good	[60]
Lyons et al.	2006	Case report	1	Good	[61]
Wullich et al.	2004	Case report	2	Fair	[62]
Schoenwaelder et al.	2004	Case report	1	Good	[63]
Tremont-Lukats et al.	2003	Case series	103	Good	[9]
Erasmus et al.	2002	Case report	1	Good	[64]
Minami, H.	2001	Case report	1	Fair	[65]
Behrens et al.	2001	Case report	1	Good	[66]
Garcia-Morales et al.	2000	Case report	1	Good	[67]
Fervenza et al.	2000	Case report	1	Good	[68]
Hayashi et al.	1998	Case report	1	Fair	[69]
Zhang et al.	1997	Case report	1	Fair	[70]
Leibman et al.	1995	Case report	2	Good	[71]
Bland et al.	1992	Case report	1	Good	[72]
Lynes et al.	1986	Case report	2	Good	[73]
Sarma et al.	1983	Case report	4	Fair	[74]

**Table 2:** Clinical manifestations and diagnosis methods in PCa patients presenting with BMs.

Author	Year of publication	Type of studies	Total sample size	All reported clinical manifestations from all the patients	Diagnostic methods	Reference
Sena et al.	2021	Case report	1	Confusion, expressive aphasia, impaired coordination of his right hand	PSA, MRI, multispectral immunofluorescence, IHC, and DNA sequencing	[36]
Pikis et al.	2021	Case series	46	Neurological symptoms	PSA	[37]
Parihar et al.	2021	Case report	1	Any neurologic or visual symptoms	PSA and PET/CT	[38]
Hung et al.	2021	Retrospective study	204	—	PSA	[39]
Son et al.	2020	Case report	1	Headache, weakness, and unintentional weight loss	PSA, brain CT, brain MRI, and bone scan	[30]
Shida et al.	2020	Case report	3	Headache, nausea, double vision, and aphasia	PSA, brain CT, and brain MRI	[29]
Saadatpour et al.	2020	Case report	1	Afebrile and unresponsive	Brain CT	[41]
Ross et al.	2020	Case report	1	No neurological symptoms	PSA, brain CT, brain MRI, and bone scan	[28]
Marchand Crety et al.	2020	Case report	1	No neurological symptoms	PSA, prostate biopsy, and brain MRI	[27]
Liu et al.	2020	Case report	1	Weakness, loss of appetite, and chronic multiple bone and joint pain	PSA, brain CT scan, brain MRI, and CSF analysis	[26]
Janda et al.	2020	Case report	1	Headache, vomiting, and decreased visual acuity	Brain CT scan, brain MRI, and PSA	[24]
Bhambhvani et al.	2020	Retrospective cohort	31	Headache, delirium, weakness, cranial nerve palsy, visual field cut, ataxia, and seizures	—	[13]
Aljarba et al.	2020	Case report	1	Headache, dizziness, and upper and lower limb weakness	Brain CT scan, brain MRI, PSA, IHC, and CSF analysis	[32]
Ahmad et al.	2020	Case report	1	Headache, right-sided facial pain, intermittent diplopia in the right eye, and partial right third nerve palsy	Brain CT, blood test, MRI, CT thorax, abdomen and pelvis, PSA, prostate biopsy, and IHC	[43]
Kosaka et al.	2019	Case report	1	Reduced motivation	PSA, brain CT scan, brain MRI, and biopsy	[23]
Kanyilmaz et al.	2019	Retrospective cohort	339	Headache and hemiparesis	PSA, brain MRI, brain CT, and histopathology	[44]
Ishizaki et al.	2019	Case report	1	Dizziness and gross hematuria	PSA, brain MRI, brain CT, and DRE	[22]
Hogan et al.	2019	Case report	1	Confusion, visual disturbances,	Brain CT scan, ETV, biopsy, and PSA	[45]
Zanatta et al.	2018	Case report	1	double vision, holocranial headache, 6th cranial nerve paresis, and 5th cranial nerve paresthesia	PSA, MRI, multispectral immunofluorescence and IHC, and DNA sequencing,	[46]
Reinas et al.	2018	Case report	1	Headache and confusion	PSA, bone scintigraphy, CT scan of the thorax, abdomen and pelvis, and MRI	[47]
Nunno et al.	2018	Case report	1	Mental status, restlessness, headache, nausea, and vomiting, gait unsteadiness, unresponsive, and mumbling incomprehensible words	Brain CT	[48]
Jack et al.	2018	Case report	1	Dizziness, nausea, vomiting, headache, diaphoresis, and hemianopsia,	PSA, MRI, and biopsy	[49]
Campagna and Feia	2018	Case report	1	Falls, memory deficits, and weight loss	PSA, brain MRI, and brain CT	[21]
Watanabe et al.	2017	Case report	1	Back pain, headache, and nausea	CT, PSA, biopsy, bone scintigraphy, contrast-enhanced CT of the chest, abdomen, and pelvis	[20]
Lam et al.	2017	Case report	2	Dysphasia	PSA, brain MRI, brain CT, and biopsy	[19]
Chang et al.	2017	Case report	1	Memory loss, delusions, and dementia	PSA, brain MRI, brain CT, IHC, and biopsy	[18]
Guraya et al.	2016	Case report	1	Confusion, altered mental status, urinary frequency, occasional incontinence, and difficulty urinating	CT, PSA, and brain MRI	[50]
Barakat et al.	2016	Case report	1	Dizziness, mild dysphagia, and imbalance	PSA, brain MRI, and brain CT	[17]
Mandaliya et al.	2015	Case report	2	Right-sided facial palsy, dysarthria, and hemiparesis,	CT scan, MRI, blood tests, and PSA	[51]
Hutton et al.	2015	Case report	1	Bony pain, lower limb weakness, collapse, increased tone, and hematuria	PSA, bone scan, MRI, and brain CT	[16]
Gajewska et al.	2015	Case report	1	—	PET/CT scan, PSA, MRI, and bone scintigraphy	[15]
Craig et al.	2015	Case report	1	Increased urinary frequency, headaches, unsteady gait, and memory loss	DRE, PSA, transrectal ultrasonography, CT, chest radiography, MRI, IHC	[14]
Hatzoglou et al.	2014	Retrospective cohort	21	—	and MRI	[10]
Placido et al.	2014	Case report	3	—	PSA and MRI	[52]
Hazell et al.	2013	Case report	4	Weakness, seizure, ataxia, back pain, confusion, headache, dysarthria, and expressive dysphasia	MRI, PSA, bone scan, and brain CT	[53]
Caffo et al.	2013	Cohort	490/9	—	Brain CT and MRI	[54]
Caffo et al.	2012	Case series	22	Headache, confusion, coma, and hyposthenia	Brain CT and MRI	[55]
Flannery et al.	2010	Case series	10	Headaches, visual deterioration, seizures, diplopia, facial weakness, and confusion	PSA and MRI	[56]
Yamada et al.	2009	Case report	1	Back pain, headache, frequent vomiting, a decreased level of consciousness, his condition deteriorated quickly	Biopsy, PSA, progastrin-releasing peptide, brain MRI, and CSF	[57]
Sweets et al.	2009	Case report	1	Palpable prostate, and grand mal seizure	PSA, abdomen and pelvis CT, prostaScint scan, brain MRI, and PAP	[58]
Kim, S. H.	2008	Case series?? Pros?	5	Headache, memory deficit, hemiparesis, visual field deficits, ataxia, dysphasia, and seizure	PSA, volumetric axial, and contrast-enhanced T1 scan	[59]
Grenader et al.	2007	Case report	1	Confusion and behavioral changes	Brain CT and MRI, PSA, and histology	[60]
Lyons et al.	2006	Case report	1	Left-sided headache, right hemiparesis, and partial epilepsy	Brain CT, cerebral angiography, and biopsy	[61]
Wullich et al.	2004	Case report	2	Neurologic alterations	PSA, brain MRI, axial T1-weighted scan, and histopathology	[62]
Schoenwaelder et al.	2004	Case report	1	Worsening headaches, mild nausea and vomiting, mild gait ataxia, brisk reflexes, and upgoing plantar reflex on the right	PSA, brain CT, MRI, and whole body bone scan	[63]
Tremont-Lukats et al.	2003	Case series	103	Headaches, delirium, memory deficits, diplopia, with III, IV, or VI CN palsies, VII and other lower CN neuropathies, hemiparesis, visual field cuts, aphasia, seizures, ataxia, and asymptomatic	Cytology, MRI, and CT myelography	[9]
Erasmus et al.	2002	Case report	1	Homonymous hemianopsia,	PSA, prostate biopsy, brain CT and MRI, and histological examination	[64]
Minami et al.	2001	Case report	1	Dysarthria and hemiplegia	PSA, intravenous pyelography and urethrocystography, brain CT, and MRI	[65]
Behrens et al.	2001	Case report	1	Personality change, forgetfulness and episodic confusion, and slow gait and inability to tandem-walk	PSA, brain MRI and CT, and histopathology	[66]
Garcia-Morales et al.	2000	Case report	1	Slight memory deficit	Bone scan, brain MRI and CT, 111In-ProstaScint scan, tomography images, and histopathology	[67]
Fervenza et al.	2000	Case report	1	Confusion, nausea, unresponsiveness, headache, unsteadiness, and mild muscle weakness	PSA and brain MRI	[68]
Hayashi et al.	1998	Case report	1	Asymptomatic	PSA, histology, and brain CT	[69]
Zhang et al.	1997	Case report	1	Transient attack of unconsciousness	PSA, biopsy, CT, and histopathology	[70]
Leibman et al.	1995	Case report	2	Rapid deterioration, dementia, rigidity and disorientation, and difficulty with walking	PSA, PAP, biopsy, brain CT and MRI, and bone scan	[71]
Bland et al.	1992	Case report	1	Seizures, left cheek pain, left orbital pain, periorbital swelling, diplopia, proptosis of the left eye, chemosis and lateral periorbital edema, poor short-term memory, diplopia, limitation of all extraocular movements of the left eye, a diminished left corneal reflex, and mild right hemiparesis.	Brain CT, histopathology, PAP, PSA, and bone scan	[72]
Lynes et al.	1986	Case report	2	Headaches, gait disturbances, incoordination, memory loss, and lethargy, lateral gaze nystagmus, papilledema, hyperactive deep tendon reflexes, and motor incoordination, and rapidly progressive dementia	Brain CT, biopsy, diethylstilbestrol/lumbar puncture, and EEG	[73]
Sarma et al.	1983	Case report	4	Left hemiplegia, hemiparesis, and continued deterioration	Brain CT	[74]

Prostate specific antigen (PSA); brain computed tomography (CT); magnetic resonance imaging (MRI); positron emission tomography (PET); cerebrospinal fluid (CSF); digital rectal examination (DRE); immunohistochemistry (IHC); endoscopic third ventriculostomy (ETV); prostatic acid phosphatase (PAP); electroencephalogram (EEG).

**Table 3:** Therapeutic approaches and prognosis status of PCa patients having BMs.

Author	Year of publication	Type of studies	Total sample size	Treatments	Prognosis	Overall survival time after the diagnosis of BM	Reference
Sena et al.	2021	Case report	1	Sipuleucel-T immunotherapy, abiraterone, prednisone, pembrolizumab, dexamethasone, craniotomy, brain-directed radiotherapy, and docetaxel chemotherapy	Passed away	—	[36]
Pikis et al.	2021	Case series	46	Prostatectomy, adjuvant radiation therapy, SRS, craniotomy, WBRT, and ADTs	Passed away	1 year and 8 months	[37]
Parihar et al.	2021	Case report	1	Docetaxel, cabazitaxel, enzalutamide, LuPSMA radioligand therapy, alpha therapy, and Ac-PSMA,	Survived	—	[38]
Hung et al.	2021	Retrospective study	204	Docetaxel, AA, enzalutamid, and cabazitaxel	—	—	[39]
Boxley et al.	2021	Retrospective study	29	ADTs, enzalutamide, abiraterone acetate, docetaxel, and cabazitaxel	—	—	[40]
Son et al.	2020	Case report	1	Bicalutamide, degarelix, leuprorelin, WBRT, chemotherapy, and craniotomy	—	—	[30]
Shida et al.	2020	Case report	3	Bicalutamide, degarelix, brachytherapy, and WBRT	3 passed away	1/3/5 months	[29]
Ross et al.	2020	Case report	1	craniotomy	Survived	—	[28]
Nguyen et al.	2020	Retrospective cohort	21	WBRT	Passed away	2/3/6 months	[42]
Marchand Crety et al.	2020	Case report	1	Hormone therapy, prostatic radiotherapy, docetaxel, and chemotherapy	Survived	—	[27]
Liu et al.	2020	Case report	1	Dexamethasone and palliative care	Passed away	5 months	[26]
Janda et al.	2020	Case report	1	Complete androgen blocking, GnRH analog, and nonsteroidal antiandrogen, chemotherapy with analgesic radiotherapy	Passed away	1 year and 8 months	[24]
Bhambhvani et al.	2020	Retrospective cohort	31	Stereotactic radiosurgery and surgical resection	Passed away	4.6/13 months	[13]
Aljarba et al.	2020	Case report	1	ADTs, surgery, and palliative care	Passed away	43 days	[32]
Ahmad et al.	2020	Case report	1	Thyroxine (steroid replacement), exposure therapy, testosterone replacement, and bicalutamide, radiotherapy	Survived	—	[43]
Kosaka et al.	2019	Case report	1	Craniotomy and ADTs, chemotherapy	—	—	[23]
Kanyilmaz et al.	2019	Retrospective cohort	339	WBRT and radiotherapy	Passed away	4.5 months	[44]
Ishizaki et al.	2019	Case report	1	ADTs and WBRT	—	—	[22]
Hogan et al.	2019	Case report	1	SRS	Survived	—	[45]
Zanatta et al.	2018	Case report	1	Cerebellopontine angle microsurgery	Survived	—	[46]
Reinas et al.	2018	Case report	1	External beam radiotherapy, bicalutamide, right parietal craniotomy, and luteinizing	—	—	[47]
Nunno et al.	2018	Case report	1	Craniotomy	—	—	[48]
Jack et al.	2018	Case report	1	Zoladex, casodex, docetaxel, lupron, ketoconazole, lupron therapy, and WBRT	Passed away	3 months	[49]
Campagna and Feia	2018	Case report	1	Craniotomy with biopsy and SRS	—	—	[21]
Watanabe et al.	2017	Case report	1	Androgen blockade therapy, denosumab, docetaxel, enzalutamide, abiraterone, and cabazitaxel	Survived	—	[20]
Lam et al.	2017	Case report	2	Near-total excision surgery	Survived	—	[19]
Chang et al.	2017	Case report	1	Dexamethasone, SRS, excision and orchiectomy, and left subcapsular orchiectomy	—	—	[18]
Guraya et al.	2016	Case report	1	Dexamethasone, steroid injection, levetiracetam, craniotomy, and radiation therapy	Passed away	12 months	[50]
Barakat et al.	2016	Case report	1	Tumor resection, SRS, craniotomy, and ADTs	Survived	—	[17]
Mandaliya et al.	2015	Case report	2	Stereotactic craniotomy, WBRT, and ADTs	Survived	—	[51]
Hutton et al.	2015	Case report	1	Dexamethasone	Passed away	—	[16]
Gajewska et al.	2015	Case report	1	Cabazitaxel	—	—	[15]
Craig et al.	2015	Case report	1	Radiotherapy, ADTs, craniotomy, dexamethasone, WBRT, bicalutamide, abiraterone, and prednisone	Passed away	—	[14]
Hatzoglou et al.	2014	Retrospective cohort	21	—	Passed away	2.8 months	[10]
Placido et al.	2014	Case report	3	Luteinizing, complete androgen blockage, bicalutamide, prostatectomy and radiation therapy, ADTs, docetaxel, abiraterone, and cabazitaxel	2 passed away, 1 survived	—	[52]
Hazell et al.	2013	Case report	4	ADTs, radiation therapy, craniotomy, WBRT, docetaxel, prednisone, and zoledronic acid	Passed away	—	[53]
Caffo et al.	2013	Cohort	9	Castration-resistantdocetaxel-based chemotherapy, and surgery	—	—	[54]
Caffo et al.	2012	Case series	22	Undergone docetaxel-based treatment, surgery, WBRT, gamma knife, chemotherapy with docetaxel and mitoxantrone, and radiotherapy	20 passed away	4 months	[55]
Flannery et al.	2010	Case series	10	ADTs, WBRT, SRS, and gamma knife	9 passed away	2–23 months	[56]
Yamada et al.	2009	Case report	1	GnRH, cisplatin plus etoposide, and combined therapy with carboplatin plus irinotecan	Survived	—	[57]
Sweets et al.	2009	Case report	1	Chemotherapy with doxorubicin, vinblastine, nilutamide, leuprolide, prostatectomy, salvage three-dimensional conformal radiotherapy, and craniotomy	Survived	—	[58]
Kim et al.	2008	Case series	5	Gamma knife SRS and WBRT	4 passed away	10 months	[59]
Grenader et al.	2007	Case report	1	Dexamethasone, phenytoin, palliative radiotherapy, antiandrogen agent, bicalutamide, subcutaneous luteinizing hormone releasing hormone agonist goserelin, zoledronic acid, and craniotomy	Survived	—	[60]
Lyons et al.	2006	Case report	1	Prostatectomy, dexamethasone, phenytoin, craniotomy and resection of tumor, and external beam radiotherapy to the brain, casodex and lupron therapy	Survived	—	[61]
Wullich et al.	2004	Case report	2	Bilateral orchiectomy, fosfestrol, transurethral resection, craniotomy, and WBRT	Passed away	1-2 month	[62]
Schoenwaelder et al.	2004	Case report	1	Dexamethasone, phenytoin, posterior fossa craniectomy, insertion of a ventriculoperitoneal shunt for hydrocephalus, oral antiandrogen therapy, and palliative radiotherapy	Passed away	4 months	[63]
Tremont-Lukats et al.	2003	Case series	103	Supportive care and SRS	Passed away	1 month	[9]
Erasmus et al.	2002	Case report	1	Hormonal therapy(cyproterone acetate), craniotomy, and radiation therapy	Passed away	—	[64]
Minami et al.	2001	Case report	1	Transurethral resection of prostate, hormone therapy with goserelin acetate and flutamide, and intermittent arterial infusion chemotherapy with cisplatin and pirarubicin	Passed away	Less than 1 month	[65]
Behrens et al.	2001	Case report	1	Dexamethasone, phenytoin, craniotomy	—	—	[66]
Garcia-Morales et al.	2000	Case report	1	chemotherapy, radiotherapy, and craniotomy	—	—	[67]
Fervenza et al.	2000	Case report	1	WBRT, SRS, craniotomy, and ADTs	Survived	—	[68]
Hayashi et al.	1998	Case report	1	Prostatectomy and craniotomy	Survived	—	[69]
Zhang et al.	1997	Case report	1	Prostatectomy, ifosfamide, local radiation therapy, and craniotomy	Passed away	19 months	[70]
Leibman et al.	1995	Case report	2	Hormonal therapy (leuprolide and flutamide) and chemotherapy (cyclophosphamide and methotrexate), and WBRT	Passed away	—	[71]
Bland et al.	1992	Case report	1	Radiation therapy and bilateral orchiectomy	Survived	—	[72]
Lynes et al.	1986	Case report	2	WBRT, suboccipital craniotomy, bilateral orchiectomy, doxorubicin hydrochloride (adriamycin), and frontal craniotomy	1 survived, 1 passed away	9 months	[73]
Sarma et al.	1983	Case report	4	Transurethral resection and bilateral orchiectomy	Passed away	—	[74]

Stereotactic radiosurgery (SRS); whole brain radiation therapy (WBRT); antiandrogen deprivation therapies (ADTs); abiraterone acetate (AA); gonadotropin-releasing hormone (GnRH).

## Data Availability

The data will be provided by the corresponding author on request.
